# Socio-economic inequalities in the multiple dimensions of access to healthcare: the case of South Africa

**DOI:** 10.1186/s12889-020-8368-7

**Published:** 2020-03-04

**Authors:** Tanja Gordon, Frederik Booysen, Josue Mbonigaba

**Affiliations:** 1 Research Impact Assessment programme (RIA), Human Sciences Research Council (HSRC), HSRC Building 134 Pretorius Street, Pretoria, 0002 South Africa; 2 School of Economic and Business Sciences (SEBS), University of Witwatersrand (Wits), Johannesburg, South Africa; 3 Department of Economics, University of KwaZulu-Natal (UKZN), Durban, South Africa

**Keywords:** Access, Health inequality, Healthcare, Concentration index, Decomposition, South Africa

## Abstract

**Background:**

The National Development Plan (NDP) strives that South Africa, by 2030, in pursuit of Universal Health Coverage (UHC) achieve a significant shift in the equity of health services provision. This paper provides a diagnosis of the extent of socio-economic inequalities in health and healthcare using an integrated conceptual framework.

**Method:**

The 2012 South African National Health and Nutrition Examination Survey (SANHANES-1), a nationally representative study, collected data on a variety of questions related to health and healthcare. A range of concentration indices were calculated for health and healthcare outcomes that fit the various dimensions on the pathway of access. A decomposition analysis was employed to determine how downstream need and access barriers contribute to upstream inequality in healthcare utilisation.

**Results:**

In terms of healthcare need, good and ill health are concentrated among the socio-economically advantaged and disadvantaged, respectively. The relatively wealthy perceived a greater desire for care than the relatively poor. However, postponement of care seeking and unmet need is concentrated among the socio-economically disadvantaged, as are difficulties with the affordability of healthcare. The socio-economic divide in the utilisation of public and private healthcare services remains stark. Those who are economically disadvantaged are less satisfied with healthcare services. Affordability and ability to pay are the main drivers of inequalities in healthcare utilisation.

**Conclusion:**

In the South African health system, the socio-economically disadvantaged are discriminated against across the continuum of access. NHI offers a means to enhance ability to pay and to address affordability, while disparities between actual and perceived need warrants investment in health literacy outreach programmes.

## Background

The United Nation’s Sustainable Development Goal (SDG) 3.8 strives towards the achievement of access to quality, effective, and affordable medical care for all and the assurance of universal coverage [1]. In addition, mandated in South Africa’s National Development Plan (NDP) is the goal to provide universal equitable, efficient and quality healthcare [2]. In light of these global and national policy prerogatives, socio-economic inequalities in access to healthcare remain high on the policy agenda.

Research finds that over one billion people in low- and middle-income countries (LMIC) are unable to afford healthcare and that the poor within these countries benefit least from healthcare utilisation [3, 4]. In the case of South Africa, the socio-economically disadvantaged are more likely to experience poor health status, disability, the simultaneous occurrence of more than one condition/disease (multi-morbidity) and are less likely to use inpatient care [5–7]. The South African health system is two-tiered with the least advantaged heavily dependent on the under-resourced public sector, while the wealthy (many of whom have private medical insurance) use the private sector [8–15]. Since 1996, user fees were waived for all seeking primary public healthcare, although eligibility for free care at public sector hospitals is subject to a means-test [16, 17]. In order to access a private healthcare facility one has to pay out-of-pocket (OOP) or be covered by health insurance (even then the patient may incur a co-payment). In 2015/16, private healthcare expenditure was 4.4% and OOP expenditure 0.06% of GDP, whereas public healthcare expenditure amounted to 4.1% of GDP and is funded from general tax [8, 17]. Although each health sector makes an almost equal contribution to GDP, the public sector services approximately 84% of the population while the private sector services a mere 16% [8, 9].

South African studies on health inequalities, however, with the exception of Harris et al. [18], are rather unidimensional in nature, generally focusing only on a limited number of outcomes rather than a wide variety of dimensions of access to healthcare. Studies tend to look at single dimensions on the pathway of access, for example, healthcare outcomes such as multi-morbidity and disability [6], life-style diseases [19, 20], child [21, 22] and maternal health [23, 24], and healthcare utilisation [7]. Current research, therefore, is limited in that it fails to examine the full spectrum of the dimensions of access. Another important point to note is that inequality in access, where it has been analysed comprehensively [18], has only been measured descriptively, whereas this study adopts a more standard method and makes use of the concentration index and employs a decomposition analysis to determine the main contributors to inequality in healthcare utilisation. As the country embarks on the implementation of National Health Insurance (NHI) [8], advancing the understanding of inequalities in access to healthcare and tracking these inequalities remains a priority.

The one purpose, therefore, of this study is to describe socio-economic inequalities in South Africans’ access to healthcare using a standardised indicator of inequality applied to an integrated conceptual framework. The other purpose is to determine how upstream need and access barriers contribute to downstream inequality in healthcare utilisation in the private and public sectors with the aid of a decomposition analysis.

### Conceptual framework

**Fig. 1:**
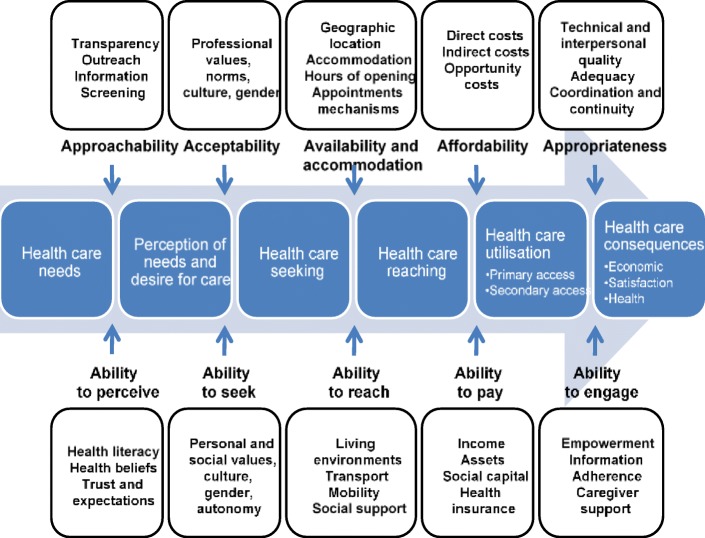
Dimensions of access to healthcare: a conceptual framework

Elsewhere, access has been defined as availability (the location of the healthcare facility and the ability of the individual to access the facility), affordability (direct/indirect costs of healthcare utilisation and the ability of the individual to meet these costs); and acceptability (the point at which the service from the provider meets the expectation of the patient) [25]. This study however, uses the even more detailed framework adopted by Levesque et al. [26] to conceptualise the various dimensions of access to healthcare (Fig. 1). These authors define access as ‘realised utilisation’. More intrinsically, access comprises the perception of an individual’s need for care, healthcare seeking, healthcare reaching and the utilisation of healthcare and its consequences. The pathway is influenced by individual and community-level health system supply-side factors: 1) approachability; 2) acceptability; 3) availability and accommodation; 4) affordability and; 5) appropriateness as well as demand-side factors: 1) ability to perceive; 2) ability to seek; 3) ability to reach; 4) ability to pay and; 5) ability to engage. Given the broad dynamics of this definition, this study uses proxies that best fit the applicable stages or dimensions of access and selected demand- and supply-side factors.

## Methods

### Data

Data analysis was conducted using the nationally representative 2012 South African National Health and Nutrition Examination Survey (SANHANES-1). The objective of the survey was to examine the current health and nutrition status of South Africans in relation to non-communicable disease (NCD) prevalence and their associated risk factors. For the purpose of the survey, 500 Enumerator Areas (EA’s) representative of the demographic profile of South Africa were identified from the 2007 HSRC Master Sample of 1000 EAs selected from the 2001 population census. Thereafter, 20 visiting points were randomly selected from each EA totalling a sample of 10,000 visiting points (VPs). Of the 10,000 households (VPs) sampled, 8168 were valid households of which 6307 (77.2%) were successfully interviewed. From the total number of valid households who consented to participate in the study, 27,580 individuals aged 15 years and older were eligible for interview. Overall, 92.6% of all qualified individuals completed the individual interview. The SANHANES-1 survey received ethical clearance from the Research Ethics Committee (REC) of the Human Science Research Council (HSRC) (REC 6/16/11/11) [27].

### Health and healthcare outcomes

Table 1 below maps out the variables selected to represent each dimension of access to healthcare based on the study’s conceptual framework (see Fig. 1).

**Table 1:** Health and healthcare outcomes, by access dimension

Access dimension	Outcome	Survey question
**Healthcare need:**		
Self-reported health (SRH)	Binary: Very good and good 1, 0 otherwise	In general how would you rate your health today? [AQ]
World Health Organisation Disability Schedule (WHODASscore)	Continuous	In the last 30 days, how much difficulty did you have in …? (12 questions) [AQ]
Kessler Psychological Distress Scale (K10)	Binary: Psychological distressed 1, 0 otherwise	The following questions concern how you have been feeling over the past 30 days. (10 questions) [AQ]
Post-Traumatic Stress Disorder (PTSD)	Binary: PTSD 1, 0 otherwise	In the past week, how much trouble have you had with the following symptoms? (17 questions) [AQ]
**Perceived healthcare need:**		
Needed care	Binary: Needed care 1, 0 otherwise	When was the last time you needed health care (from a doctor or hospital)? [AQ]
**Healthcare seeking:**		
Household healthcare postponed	Binary: Household healthcare postponed 1, 0 otherwise	In the last 12 months, have you put off or postponed getting the healthcare you need? [VPQ]
**Availability:**		
Household distance to a healthcare facility	Binary: 0–10 Km away from a healthcare facility 1, 0 otherwise	How far do you live from the nearest health clinic or hospital? [VPQ]
**Healthcare reaching:**		
Unmet need	Binary: Unmet need 1, 0 otherwise	The last time you needed health care, did you get health care? [AQ]
**Affordability:**		
Household difficulty affording cost of care	Binary: Yes 1, 0 otherwise	In the past 12 months, have you had difficulty affording the cost of necessary medical care? [VPQ]
Household difficulty affording prescription medicine	Binary: Yes 1, 0 otherwise	In the past 12 months, have you had difficulty affording the cost of prescription medication? [VPQ]
**Ability to pay:**		
Household private medical insurance	Binary: In my own name/ through a family member 1, 0 otherwise	Do you have private medical aid/ health insurance either in your own name or through another family member? [VPQ]
**Healthcare utilisation:**		
Household private care	Binary: Private 1, 0 otherwise	Where do you usually get your healthcare from? [VPQ]
Household public care	Binary: Public 1, 0 otherwise	
Individual private care	Binary: Private doctor/hospital/clinic in the last year 1, 0 otherwise	When was the last time that you received health care from a private doctor/hospital/clinic? [AQ]
Individual public care	Binary: Public doctor/hospital in the last year 1, 0 otherwise	When was the last time that you received health care from a public doctor/hospital/clinic? [AQ]
Overall individual care	Binary: Individual private or public care in the last year 1, 0 otherwise	
**Healthcare consequences:**		
Healthcare service satisfaction	Binary: Very satisfied and satisfied, 0 otherwise	In general, how satisfied were you with how the health care services were run in your area? [AQ]
Healthcare service provider satisfaction	Binary: Very satisfied and satisfied, 0 otherwise	How would you rate the way health was provided in your area? [AQ]

*AQ* adult individual questionnaire, *VPQ* visiting point household questionnaire

### Wealth index

To investigate the socio-economic gradient in each of the health and healthcare outcomes in the access pathway, a wealth index and corresponding wealth quintiles were constructed by applying Multiple Correspondence Analysis (MCA) to the household survey data. Use was made of a total of 16 variables, including housing type, water and sanitation services, and ownership of 13 household assets. The percentage inertia explained by the first dimension is approximately 90%. The wealth index was used as it is considered a more reliable measure of socio-economic status (SES) in developing countries as compared to income [28].

### The concentration index

The concentration curve plots the cumulative proportion of the population by SES, beginning with the least advantaged and ending with the most advantaged, against the cumulative proportion of health or ill health. The line of equality or the diagonal signifies the absence of inequality. If the curve lies above the line, ill health falls on the least advantaged in the population, and if it lies below, the more advantaged. The further the curve lies from the diagonal the greater the degree of inequality. The concentration index is defined as twice the area between the curve and the line of equality. It takes on a positive value when it lies below the line of equality and a negative value when it lies above. A positive value can be interpreted as the concentration of health among the relatively wealthy and a negative value among the relatively poor. The minimum value the index can take is − 1 and the maximum value is + 1. Should the index be equal to zero (or not statistically significantly different from zero), no inequality exists [29–31].

According to the literature, the standardised concentration index is suitable for variables with a ratio scale, the equation of which is as follows:

1 $$ C=\frac{2}{\mu}\mathit{\operatorname{cov}}\left(h,r\right) $$

where *C* is the standardised concentration index, *h* is the healthcare variable, *μ* is the mean of the healthcare variable, and *r* is the ith- ranked individual in the socio-economic distribution from the relatively poorest to the richest [28, 29, 31, 32].

Bounded variables, on the other hand, complicate the measurement of inequality. Given that bounded variables can take the form of attainments or short falls the mirror property that requires absolute values of health *I*(*h*) and ill health *I*(1 − *h*) to be equal with different signs, is not satisfied with the standardised concentration index [32]. In this regard, one common practice concerning variables with a limit is the use of the Erregyer corrected concentration index. The index is desirable as it satisfies properties required for bounded variables [33]. The equation for the Erregyer index is as follows:

2 $$ CCI=\frac{4\mu }{b-a}\ast C $$

where *CCI* is the corrected concentration index, *μ* is the mean of the attained healthcare, *b* and *a* the maximum and minimum values, respectively, and *C* the standardised concentration index [32–34].

### Decomposition analysis

A decomposition analysis was conducted to determine how upstream factors such as health status, need and access barriers contribute to downstream socio-economic inequality in healthcare utilisation. Following Wagstaff [35], Eq. 3 depicts the linear relationship between the health variable (utilisation) and its determinants:

3 $$ {h}_i={\beta}_0\sum \limits_{k=1}^K{\beta}_k{x}_{ik}+{\varepsilon}_i $$

where *h*_*i*_ is the healthcare variable of interest, *x*_*ik*_ the set of demographic and socio-economic contributing factors, and *ε*_*i*_ the error term. Like the concentration indices, the decomposition technique used for the standard concentration index (C) (not shown here) [35–37] is modified to suit the corrected concentration index (CCI) as follow:

4 $$ CCI(h)=4\left[\sum \limits_{k=1}^K{\beta}_k{\overline{x}}_kC\left({x}_k\right)+G{C}_{\varepsilon}\right] $$

The decomposed *CCI* is the summed product of the degree of responsiveness, i.e. the elasticity $\left({\beta}_k{\overline{x}}_k\right)$ to health changes and the degree of socio-economic inequality *C*(*x*_*k*_) in that determinant, plus the generalised concentration index of the error term (*GC*_*ε*_), all multiplied by 4. All things being equal, a positive contribution (*x* %  > 0) by a factor would decrease socio-economic inequality. Alternatively, a negative contribution (*x* %  < 0), all things being equal, would increase socio-economic inequality [20, 38, 39]. The unexplained part of the contribution of factors to inequality, the residual, can take on negative values, with an explained percentage in excess of 100%, which, by interpretation, suggests that measured inequality is completely explained by the model’s explanatory variables [40], as has been the case in other decomposition studies [40–44]. To determine whether actual and perceived need and access barriers are sector-specific, the decomposition analysis was stratified by private/public healthcare utilisation as characterised by the two-tiered South African health system. The Generalised Linear Model (GLM) from the binomial family with a link function was used as it is considered the least sensitive to the choice of reference group when the dependent variable is a binary health outcome [45]. The decomposition analysis was bootstrapped at 500 replications to obtain standard errors and *p*-values for the statistical significance of the absolute contributions [46]. Data analysis was conducted in STATA software version 15 and weighted with post stratified sample weights.

## Results

### Description

Table 2 describes the adult sample’s socio-demographic characteristics and each of the access variables. The adult sample comprised slightly more females than males (52% versus 48%). The average age of respondents was 37 years. Respondents mainly comprised Africans (78%) and lived mainly in urban areas (67%).

**Table 2:** Summary statistics

Variable	Mean (%)	SE	n
**A. Demographics**			
**Sex:**			
Male	47.96	0.004	15,911
Female	52.04	0.004	15,911
**Age:**			
Age	36.75	0.128	15,886
**Race:**			
African	77.64	0.003	15,839
non-African	22.36	0.003	15,839
**Geographical area:**			
Urban	66.70	0.004	15,405
Rural	33.30	0.004	15,405
**B. Access dimension**			
**Healthcare need:**			
Self-reported health	78.49	0.003	14,351
WHODAS score	5.29	0.096	13,407
Psychological distress	6.46	0.002	14,215
Perceived healthcare need:			
Needed care	50.57	0.005	9937
**Healthcare seeking***:*			
Household healthcare postponed	21.19	0.005	5651
**Availability:**			
Household distance to a healthcare facility	77.46	0.005	5817
**Healthcare reaching:**			
Unmet need	3.16	0.002	6852
**Affordability:**			
Household difficulty affording cost of care	27.64	0.006	5613
Household difficulty affording prescription medicine	26.09	0.006	5603
**Ability to pay:**			
Household private medical insurance	21.09	0.005	5804
**Healthcare utilisation:**			
Household private care	27.38	0.006	5823
Household public care	71.32	0.006	5823
Individual private care	30.52	0.004	11,029
Individual public care	42.37	0.005	10,489
Overall individual care	59.49	0.005	10,293
**Healthcare consequences:**			
Healthcare service satisfaction	71.37	0.004	14,143
Healthcare service dissatisfaction	69.35	0.004	14,059

Note: All estimates are weighted proportions, *SE* Standard error, *WHODAS score* World Health Organisation Disability Assessment Schedule, *K10* Kessler Psychological Distress Scale

Overall, 78% of individuals self-reported good or very good health. On average, 5% of individual respondents found it difficult to complete basic physical, cognitive and social activities. In addition, 6% of respondents experienced high or very high levels of psychological distress. From the results, just over 50% of the population received the healthcare they required and just about 21% of households postponed seeking healthcare. Unmet need was low, at 3%, and just over three quarters of households lived within 10 km from a healthcare facility. Roughly 21% of households had private medical insurance. In addition, an estimated 28% of households had difficulty affording their medical care and 26% their prescription medication. Among individual respondents, 31% used private care and 42% public care in the year prior to the survey, with 59% having used either a private or public healthcare facility. Approximately seven in ten households used a public healthcare facility compared to only 27% of households that used a private facility. In terms of satisfaction, 71 and 69% of respondents were satisfied or very satisfied with their healthcare services and service provider, respectively. These averages, however, mask substantial socio-economic inequalities, as illustrated by the patterns across the wealth quintiles (Table 3) and the estimates of the concentration indices (Table 4).

**Table 3:** Health and healthcare outcomes in each access dimension, by wealth quintile

Access dimension	Quintile 1 (%)	Quintile 2 (%)	Quintile 3 (%)	Quintile 4 (%)	Quintile 5 (%)	F-statistic	***p***-value
**Healthcare need:**							
Self-reported health	74.52	75.98	75.94	78.47	83.42	20.1	0.000
WHODAS score	6.10	6.09	5.65	5.00	3.74	20.7	0.000
Psychological distress	8.48	6.87	8.06	6.92	2.99	21.9	0.000
**Perceived healthcare need:**							
Needed care	49.00	45.45	46.78	53.72	54.54	12.0	0.000
**Healthcare seeking:**							
Household healthcare postponed	28.88	26.21	23.22	15.19	10.65	39.4	0.000
**Availability:**							
Household distance to a healthcare facility	61.75	73.47	80.15	86.53	86.64	73.4	0.000
**Healthcare reaching:**							
Unmet need	5.55	3.80	2.96	3.26	1.59	7.9	0.000
**Affordability:**							
Household difficulty affording cost of care	36.45	31.47	29.38	24.32	15.22	36.1	0.000
Household difficulty affording prescription medicine	34.01	31.83	26.85	22.99	12.61	41.4	0.000
**Ability to pay:**							
Household private medical insurance	3.01	3.69	10.73	23.50	66.53	683.7	0.000
**Healthcare utilisation:**							
Household private care	8.01	10.09	16.44	32.75	70.92	513.5	0.000
Household public care	88.47	88.70	82.29	65.75	30.05	430.1	0.000
Individual private care	19.85	18.62	25.02	34.30	48.26	153.8	0.000
Individual public care	52.39	50.36	46.97	42.81	24.18	108.2	0.000
Overall individual care	59.13	56.73	57.25	60.65	62.18	4.1	0.003
**Healthcare consequences:**							
Healthcare service satisfaction	70.77	68.34	66.81	68.35	79.91	38.6	0.000
Healthcare service provider satisfaction	69.25	66.25	66.13	64.20	79.41	49.5	0.000

Note: All estimates are weighted proportions; *WHODAS score* World Health Organisation Disability Assessment Schedule, *K10* Kessler Psychological Distress Scale

**Table 4:** Socio-economic inequality in access to healthcare, by dimension

Access dimension	C/CCI	SE	***p-***value
**Healthcare need:**			
Self-reported health	0.074	0.020	0.000
WHODAS score	−0.101	0.025	0.000
Psychological distress	−0.041	0.008	0.000
**Perceived healthcare need:**			
Needed care	0.060	0.026	0.022
**Healthcare seeking:**			
Household healthcare postponed	−0.154	0.013	0.000
**Availability:**			
Household distance to a healthcare facility	0.210	0.013	0.000
**Healthcare reaching:**			
Unmet need	−0.029	0.008	0.000
**Affordability:**			
Household difficulty affording cost of care	−0.162	0.014	0.000
Household difficulty affording prescription medicine	−0.169	0.014	0.000
**Ability to pay:**			
Household private medical insurance	0.490	0.011	0.000
**Healthcare utilisation:**			
Household private care	0.490	0.012	0.000
Household public care	−0.462	0.013	0.000
Individual private care	0.247	0.026	0.000
Individual public care	−0.231	0.027	0.000
Overall individual care	0.033	0.029	0.257
**Healthcare consequences:**			
Healthcare service satisfaction	0.074	0.028	0.008
Healthcare service provider satisfaction	0.078	0.028	0.006

Note: *C* Standard concentration index, *CCI* Erregyer corrected concentration index, *SE* Standard error, *WHODAS score* World Health Organisation Disability Assessment Schedule, *K10* Kessler Psychological Distress Scale

### Socio-economic inequalities in access to healthcare

#### Healthcare need and perceived healthcare need

Table 4 shows the concentration index for good self-reported health to be positive in value and statistically significant in margin. That is, relatively wealthier individuals perceived their current health state as very good or good (CCI + 0.074, *p* < 0.001). Concentration indices for respondents who had difficulty completing physical, cognitive and social tasks (C − 0.101, *p* < 0.001) or reported psychological distress (CCI − 0.041, *p* < 0.001) lie below zero. In other words, the socio-economically disadvantaged are more likely to have poor health outcomes. In terms of perceived healthcare need, relatively economically better-off respondents were more likely to perceive a need for healthcare (CCI + 0.060, *p* = 0.022).

#### Healthcare seeking and reaching

**Fig. 2:**
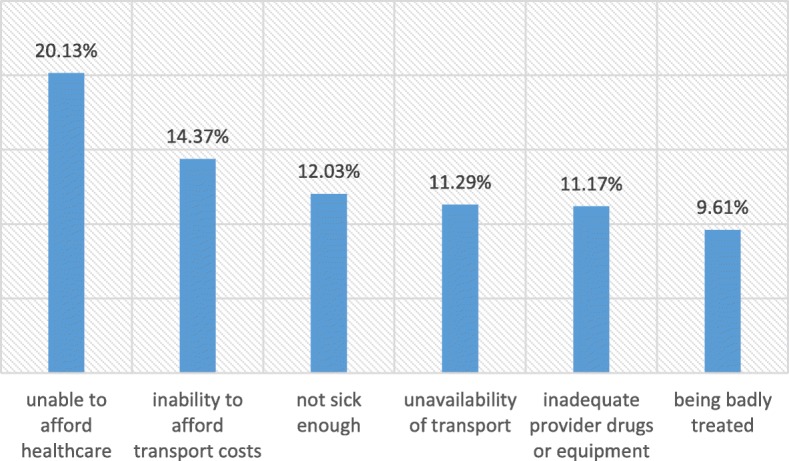
Most common reasons for households postponing healthcare

Socio-economically disadvantaged households were more likely to postpone seeking healthcare compared to those at an advantage (CCI − 0.154, *p* < 0.001). Relatively wealthy households were more likely to be located within a 10 km radius of a healthcare facility in comparison to relatively poorer households (CCI + 0.210, *p* < 0.001). From Fig. 2, the most common reason households postponed obtaining healthcare was because they could not afford care, followed by high transportation costs. The socio-economically disadvantaged were also more likely than those at an advantage to need healthcare but to report not receiving care (CCI − 0.029, *p* < 0.001).

#### Affordability, healthcare utilisation and healthcare consequences

In terms of affordability and ability to pay, which provides a bridge between reaching and using healthcare [26], results show households at an economic advantage to be more likely to have private medical insurance when compared to those at a socio-economic disadvantage (CCI + 0.490, *p* < 0.001). Economically disadvantaged households found it difficult to pay for their medical care (CI − 0.162, *p* < 0.001) and prescription medicine (CI − 0.169, *p* < 0.001). Although individual overall utilisation was unequally distributed across the five wealth quintiles, the summary measure of inequality was not significantly different from zero (CCI + 0.033, *p* = 0.257) and hence overall utilisation was not decomposed. The concentration indices depicted in Table 4 also differentiate the private and public sectors, respectively, in terms of the nature of healthcare utilisation. Private care (CCI + 0.247, *p* < 0.001) was concentrated among relatively better-off individuals, while those individuals who were economically worse-off depended on the public sector (CCI − 0.231, *p* < 0.001). Sector-specific household-level socio-economic inequalities were even more pronounced, with concentration indices as high as CCI + 0.490 (*p* < 0.001) for private healthcare and CCI − 0.462 (*p* < 0.001) for public healthcare utilisation. In terms of healthcare consequences, the results show that relatively wealthy individuals were more likely to report being satisfied or very satisfied with their healthcare service (CI + 0.074, *p* = 0.008) and service provider (CI + 0.078, *p* = 0.006), respectively.

#### Decomposition of socio-economic inequality in healthcare utilisation

Table 5 shows the results of the decomposition analysis. The columns report the margins, absolute contributions (the product of each determinant’s elasticity and CI) and their bootstrapped standard errors and *p*-values, as well as the percentage contributions of each explanatory factor. In terms of sector-based healthcare utilisation, two factors, namely household wealth (45.20%) and access to private medical insurance (46.40%), together explained almost all of the observed inequality in private sector healthcare utilisation. The same two factors (household wealth – 34.76% and private medical insurance – 48.58%), together with being African (20.24%), were all statistically significant and large contributors to inequality in public sector healthcare utilisation. Subjectively perceived need (12.81%, *p* = 0.001), and challenges with the affordability of care (− 6.62%, *p* = 0.008) made modest but statistically significant contributions to inequality in private sector healthcare utilisation. Need also made a modest (− 12.44%) but statistically significant (*p* = 0.002) contribution to public sector healthcare utilisation. For private sector healthcare utilisation, the contribution of age was statistically significant (*p* = 0.004), but small (1.96%). In the case of public sector healthcare utilisation, the contribution of self-reported health was small (2.12%) yet statistically significant (*p* = 0.001). The unexplained residuals for both the private (− 11.13) and public (− 0.48) decomposition models are negative and, as a result, the need, access and other variables explain all of the measured inequality in healthcare utilisation.

**Table 5:** Decomposition analysis of private and public individual healthcare utilisation

	Individual healthcare utilisation											
	Private care						Public care					
**Variable**	**Margins**	**Absolute**	**SE**	***p*-value**	**(%)**	**Total**	**Margins**	**Absolute**	**SE**	***p*-value**	**(%)**	**Total**
**Sex:**												
Male = reference												
Female	−0.025	0.001	0.001	0.321	0.31	0.31	0.071^a^	−0.002	0.001	0.094	0.92	0.92
**Age**	0.001^a^	0.005	0.001	0.004	1.96	1.96	0.001^c^	0.003	0.001	0.085	−1.24	−1.24
**Race:**												
Non-African = reference												
African	−0.006	0.003	0.007	0.767	1.03	1.03	0.108^a^	−0.047	0.005	0.000	20.24	20.24
**Geographical area:**												
Rural = reference												
Urban	0.027	0.015	0.009	0.181	5.95	5.95	−0.029^c^	−0.016	0.005	0.081	6.78	6.78
**Self-reported health:**												
Poor health = reference												
Good health	−0.029	−0.002	0.001	0.148	−0.89	−0.89	− 0.065^a^	−0.005	0.001	0.001	2.12	2.12
**WHODAS score**	−0.001	0.002	0.001	0.332	0.66	0.66	0.002	−0.004	0.002	0.167	1.54	1.54
**Psychological distress:**												
Not distressed = reference												
Distressed	0.041	−0.002	0.001	0.305	−0.67	−0.67	0.024	−0.001	0.001	0.509	0.42	0.42
**Needed care:**												
No = reference												
Yes	0.519^a^	0.032	0.008	0.001	12.81	12.81	0.472^a^	0.029	0.005	0.002	−12.44	−12.44
**Household healthcare postponed:**												
No = reference												
Yes	−0.028	0.005	0.004	0.381	1.95	1.95	0.007	−0.001	0.002	0.754	0.56	0.56
**Unmet need:**												
No = reference												
Yes	0.015	0.000	0.001	0.763	−0.18	−0.18	−0.042	0.001	0.001	0.537	−0.52	−0.52
**Household distance to a healthcare facility:**												
More than 10Km away = reference												
0–10 Km away	0.023	0.005	0.004	0.273	2.02	2.02	0.008	0.002	0.002	0.635	−0.75	−0.75
**Household medical insurance:**												
No = reference												
Yes	0.208^a^	0.115	0.010	0.000	46.40	46.40	−0.204^a^	− 0.112	0.008	0.000	48.58	48.58
**Household difficulty affording cost of care:**												
No = reference												
Yes	0.083^a^	−0.016	0.005	0.008	−6.62	−6.62	−0.034	0.007	0.003	0.197	−2.89	−2.89
**Household difficulty affording prescription medicine:**												
No = reference												
Yes	−0.014	0.003	0.006	0.663	1.20	1.20	0.027	−0.006	0.003	0.291	2.41	2.41
**Wealth index:**												
Quintile 1 = reference												
Quintile 2	−0.007	0.002	0.008	0.822	0.89		0.041^c^	−0.014	0.004	0.059	5.83	
Quintile 3	0.052^c^	−0.004	0.002	0.086	−1.47		−0.013	0.001	0.001	0.589	−0.38	
Quintile 4	0.075^b^	0.020	0.007	0.011	8.19		−0.035	− 0.010	0.004	0.138	4.15	
Quintile 5	0.130^a^	0.093	0.021	0.000	37.58	45.20	−0.081^b^	− 0.058	0.013	0.007	25.16	34.76
**Residual**		−0.027				−11.13		0.001				−0.48
**Total**		0.247				100.00		−0.231				100.00

Note: *SE* Standard error, *%* Percentage contribution, *WHODAS score* World Health Organisation Disability Assessment Schedule, *K10* Kessler Psychological Distress Scale, *PTSD* Post-Traumatic Stress Disorder; ^a^statistically significant at the 1% level; ^b^ statistically significant at the 5% level; ^c^statistically significant at the 10% level

## Discussion

Levesque et al. [26] provide an in-depth conceptualisation of the term access to healthcare. In essence, a pathway is described beginning with healthcare need, followed by perceived healthcare, healthcare seeking, healthcare reaching, healthcare utilisation and lastly healthcare consequences. This paper provides an exposition of socio-economic inequalities across this continuum of access using a set of 17 indicators.

All three measures of health status used in the analysis exhibited a socio-economic gradient, with healthcare need (poorer health status) concentrated in the poor. Another study also found that those socio-economically disadvantaged were most likely to report disability in relation to their intellect and emotions [5]. Concerning psychological distress, other studies also have found a lower prevalence among individuals with high incomes groups compared to those who belong to low income groups [47–49].

The ability to identify one’s healthcare needs is the next stage along the pathway of access to healthcare [26]. In SANHANES-1, respondents reported when last they needed healthcare. Financially better-off respondents were more likely to perceive a subjective need for healthcare. The fact that need was concentrated in the poor, but that subjectively perceived need for healthcare was concentrated among those who were better off, is of concern. In terms of the ability to perceive one’s needs [26], this disparity highlights the potential importance of health literacy in addressing health beliefs that are barriers to healthcare seeking [50]. Where approachability may be the problem [26], community-based outreach through ward-based teams of community health workers may provide a means for enhancing access [51].

In the matter of seeking care, relatively poorer households sometimes postponed obtaining healthcare. The most common reason households gave for not seeking care was their inability to afford healthcare. McLaren et al. [52] also found both monetary and time travel costs constrained an individual’s healthcare seeking behaviour. Harris et al. [18] instead, found the most common reason for postponed care was that respondents considered themselves not sick enough to seek treatment, exemplified here in the pro-rich inequality in subjectively perceived need for healthcare.

Access involves more than just the first contact a patient has with a health facility [26]. Findings from this study show the socio-economically disadvantaged to be more likely to have expressed an unmet need for healthcare. Allin and Masseria in their study on European countries found those with lower incomes and poorer health were also more likely to report unmet need [53]. Seeing that financially better-off households were more likely to live within a 10 km radius of a facility, availability may be an important supply-side constraint in regards to the greater occurrence among the poor of postponed care and unmet need. Cabieses and Philippa refer to access barriers of this nature as physical or geographical barriers [54]. In lieu of expanding healthcare infrastructure in the long term, extended opening hours may help address these barriers to access in settings with high patient volumes, as may be the provision of free or subsidised patient transport.

Once an individual realises he/she has a healthcare need, is able to perceive their need, seek and reach healthcare, utilisation takes places [26]. Noteworthy in this study is the expected high magnitude of concentration in the public and private sectors by the poor and the wealthy, respectively, which provides further evidence of the divide between the public and private healthcare sectors in the two-tiered South African healthcare system [9–12]. These inequalities in utilisation are attributable to the substantial socio-economic gradients reported in affordability (difficulty with affording the cost of care and medicine), and especially in ability to pay (access to private medical insurance). Literature on the full spectrum of inequality in access to healthcare as described in this study may be scant but there are studies that consider socio-economic inequalities between the public and private healthcare sectors. One such study in Mongolia found private hospital outpatient visits and inpatient admissions were concentrated among those economically better-off while the worse-off used public secondary outpatient care [55]. Saito et al. [41] instead made an overall comparison between sectors in Nepal and found significant pro-rich inequality in private healthcare use but found no conclusive evidence for inequality in public healthcare use.

The final stage on the pathway includes healthcare outcomes or the consequences of service use [26]. Patients’ self-reported assessment of service quality is subjective and presents with it a number of limitations [56]; nonetheless, the patient has an opportunity to give feedback on their overall healthcare experience. From the descriptive results, the study finds high satisfaction levels with healthcare. Similarly, other researchers have reported high levels of satisfaction in nationally representative surveys [57–59]. Conversely, greater dissatisfaction has been reported among patients who are disadvantaged socio-economically [57, 58, 60]. Findings from other research show that over a third of patients who used a public facility were dissatisfied with the quality of care they received compared to the small proportion of patients who received private care [18]. Despite this public-private divide in satisfaction, one study, however, found that SES still predicts patient satisfaction even after adjusting for facility type [58]. The Ideal Clinic programme offers a means to improve the quality of public primary healthcare services that is the first port of call for the majority of South Africans [61].

In line with findings from other African countries [62], wealth was found to be one of the highest contributors to inequality in healthcare utilisation. Private medical insurance has been considered an important determinant of access to healthcare in South Africa, that is, those with healthcare cover are not exempted from but face lower odds of financial impoverishment due to exorbitant healthcare costs [18, 63]. Ability to pay, proxied by household wealth and access to private medical insurance, and race, which, in South Africa’s case remains indicative of socio-economic status, explain almost all of the inequality in healthcare utilisation. Resonating with findings in this paper, other studies also find health insurance as a major contributing factor to inequality in access to healthcare [64, 65]. The proposed NHI scheme, which comprises a single-payer fund purchasing services from public and private sector service providers, if affordable and effectively implemented, may provide one lever for enhancing South African’s ability to pay for healthcare, while its capacity for strategic purchasing may assist in addressing affordability concerns, especially in the private sector. The continued improvement of the economic circumstances of the poor presents a second important lever for improving the poor’s access to healthcare.

Only one other study has conducted a sector-specific decomposition analysis of inequalities in healthcare use, this in Nepal [41]. The authors, using a much smaller set of explanatory variables, which apart from need excludes upstream proxies of other pathways on the access continuum, detect some differences in the factors contributing to inequality in public as opposed to private healthcare use. Age and education matter substantially more in explaining public than private sector inequality. Self-reported disease, at more than 50%, and household consumption, at around 88%, matter considerably but relatively equally for inequality in healthcare use in the public and private sectors. Need therefore matters much more in the Nepal setting than in the South African setting, but proxies of socioeconomic status more or less equally. Similar to our study, the unexplained residual is substantially larger for private than public healthcare [41].

The study has a number of limitations. The operationalisation of the conceptual framework is entirely dependent on the specific nature of the data available from the survey employed in the analysis, which precludes the analysis from being a perfect representation of the full dynamics of the access pathway. Nevertheless, this study does encompass indicators of each of the framework’s core dimensions and a selection of the supply- and demand-side factors, thus presenting a more nuanced and complete perspective on the far-reaching and inter-related nature of socio-economic inequalities in health and healthcare in South Africa than that available from other studies. The variability of self-reported data present another limitation to the study. Self-reported data is largely dependent on the cognitive ability and socio-demographic characteristics of the respondent [66, 67]. So for example, concentration among the relatively wealthy of their better assessment of healthcare needs may simply be a function of their greater levels of education. There was considerable non-response in the survey. The results, therefore, are indicative rather than fully representative of the situation in South Africa. Recall bias, in addition adds to the possible bias of subjectivity and reliability of patient-reports [66]. Lastly, the data used in the analysis of this study is dated and may not account for any recent scale-up of healthcare facilities or other shifts in the health system and its environment. It is necessary, therefore, that health authorities consider commissioning SANHANES-2 to enable researchers to assess progress on these entrenched inequalities in access and to set a pre-NHI baseline.

## Conclusion

Papers that examine the full spectrum of the dimensions of access to healthcare are important diagnostic tools to inform health policy. The intended purpose of this study was to measure inequality in access to healthcare, along a multi-dimensional pathway. According to the results, the poor are disadvantaged across all dimensions of the access pathway. Constraints on affordability, and, predominantly, ability to pay, are the main drivers of inequality in healthcare use. NHI offers a means to enhance ability to pay and to address affordability, while disparities between actual and perceived need warrants investment in health literacy outreach programmes.

## Data Availability

The data analysed is available on reasonable request from the HSRC.

## Abbreviations

C: Standard concentration index
CCI: Erregyer corrected concentration index
EA: Enumerator Area
GDP: Gross Domestic Product
GLM: Generalised Linear Model
HSRC: Human Science Research Council
K10: Kessler Psychological Distress Scale
LMIC: Low- and Middle-Income Countries
MCA: Multiple Correspondence Analysis
NCD: Non-Communicable Diseases
NDP: National Development Plan
NHI: National Health Insurance
OECD: Organisation for Economic Co-operation and Development
OOP: Out of Pocket
PHC: Primary Healthcare
SANHANES: South African National Health and Nutrition Examination Survey
SDG: Sustainable Development Goals
SDH: Social Determinants of Health
SES: Socio-Economic Status
UHC: Universal Health Coverage
VP: Visiting Point
WHODASscore: World Health Organisation Assessment Schedule
